# Surgical tracheostomy in COVID-19 patients: report of 5 cases

**DOI:** 10.11604/pamj.supp.2020.35.2.23615

**Published:** 2020-05-29

**Authors:** Ouissal Aissaoui, Afak Nsiri, Mohamed Anass Fehdi, Mohamed Mouhaoui, Rachid Alharrar

**Affiliations:** 1 Anesthesiology and Intensive Care Department, University Hospital of Casablanca, Casablanca, Morocco

**Keywords:** Surgical tracheostomy, health care worker´s safety, COVID-19

## Abstract

Severe acute respiratory distress syndrome (ARDS) related to SARS-COV-2 is resulting in increasing numbers of patients requiring mechanical ventilation. Although tracheostomy may reduce the duration of mechanical ventilation in these patients, it is considered a highly aerosol generating procedure and controversies regarding its safety, time of realization and indications remain to date. We share our experience about 5 cases of surgical tracheostomy in COVID-19 patients performed in our ICU.

## Introduction

Coronavirus disease 2019 (COVID-19) is a worldwide pandemic, with over 3 million cases diagnosed to date. Morocco counts more than 4500 cases and more than 150 deaths. Our university hospital has 3 COVID-19 ICUs dedicated to severe and critical cases. In our ICU, we cared for 20 patients to date. Mechanical ventilation was necessary in 8 cases. The pandemic has resulted in an increasing number of patients requiring endotracheal intubation and prolonged ventilator support. Tracheostomy may reduce the duration of mechanical ventilation, however, it is considered to be a highly aerosol generating procedure and the controversy around its indications and time of realization still remains. We share our experience about a series of 5 cases of surgical tracheostomies in COVID-19 patients with prolonged mechanical ventilation.

## Patients and observation

**Case 1:** a 70 years old male patient, with no co-morbidities was admitted to our ICU for acute respiratory distress syndrome (ARDS) due to SARS-COV-2 infection identified by rt PCR on nasopharyngeal swab. Chest computed tomography identified bilateral ground glass opacities with a crazy paving aspect. On admission, he was conscious, with a GCS at 15/15, he had a respiratory rate of 34 cycle per minute, a pulse oximetry at 85%. Blood pressure was 150/80mmHg and heart rate 136 beat per minute. Capillary glycemia was at 1,2g/L and temperature was 38.8°C. Oxygen therapy was initiated using a high concentration mask (12l/min) and venous access was established. Specific treatment associating hydroxychloroquine and azithromycin was administrated. Arterial blood exam showed respiratory alkalosis with hypoxemia (PO2 = 40mmHg). Blood tests showed hyperferritinemia (3350ng/ml), elevated CRP (520mg/l) and lymphopenia at 360E/mm^3^. Two days later, the patient was intubated and protective mechanical ventilation was started. After 7 days of mechanical ventilation, extubation was carried out. Extubation was unsuccessful and the patient required re-intubation after 6 days. Surgical tracheostomy was performed on day 18 days of hospitalization, after 25 days of symptom´s onset and after a total of 13 days of mechanical ventilation. The patient died 10 days later due to multi-organ failure secondary to cytokine storm. SARS-COV-2 remained positive.

**Case 2:** an 82 years old female patient, with a known history of hypertension was admitted for ARDS due to SARS-COV-2 infection identified by rt PCR on nasopharyngeal swab. The patient was confused with a GCS at 14/15, respiratory rate was 40 cycle per minute, pulse oximetry was 80% with cyanosis and sweating. Blood pressure was 110/60mmHg and heart rate 145 beat per minute. Capillary glycemia was at 1,8g/L and temperature was 38.5°C. The patient was immediately intubated then protective mechanical ventilation was initiated. Central venous access was inserted. Urinary catheter and gastric tube were placed. Specific treatment associating hydroxychloroquine and azithromycin was administrated and heavy sedation was initiated. Blood tests showed lymphopenia (320E/mm^3^), hyperferritinemia (3530ng/ml), elevated CRP (450mg/l), elevated fibrinogen levels (8.2g/l). Chest computed tomography identified bilateral ground glass opacities. Prolonged ventilation and weaning were anticipated (age, poor neurological status), so tracheostomy was performed after 14 days of mechanical ventilation and 23 days of symptoms. The patient died later on due to septic shock secondary to nosocomial multi-drug resistant pulmonary infection. SARS-COV-2 PCR remained positive in this case.

**Case 3:** a 53 years old, with no co-morbidities was admitted into our ICU for ARDS due to SARS-COV-2 infection identified by rt PCR on nasopharyngeal swab. His chest computed tomography identified bilateral ground glass opacities with images of consolidation. He was conscious with a GCS at 15/15, respiratory rate was 30 cycle per minute, pulse oximetry was 94%. Blood pressure and heart rate were normal. Oxygen therapy was initiated using a high-concentration mask and venous access was secured. Blood tests showed hyperferritinemia (3700ng/ml), elevated CRP (420mg/l), lymphopenia (600E/mm^3^) and elevated fibrinogen levels (7,5g/l). He was intubated 2 days later due to hypoxia and hypercapnia, protective mechanical ventilation along with heavy sedation was carried on. He was extubated after 8 days. Extubation was unsuccessful and the patient was re-intubated after 5 days. Tracheostomy was performed 22 days after the onset of symptoms and after 13 days of mechanical ventilation. The patient is still on ventilator support to date. SARS-COV-2 PCR was positive when we performed tracheostomy, it turned negative 7 days later.

**Case 4:** a 49 years old female patient, with history of breast cancer under chemotherapy, was admitted to our ICU for hypoxemic pneumonia due to SARS-COV-2 infection. Chest computed tomography showed bilateral ground glass opacities with a crazy paving aspect. On admission, the patient was conscious, with a GCS at 15/15, respiratory rate was 30 cycle per minute, pulse oximetry was 80%. Oxygen therapy using a high-concentration mask was initiated and peripheral intravenous line secured. The association hydroxychloroquine- azithromycin was administrated. Blood tests showed lymphopenia 550E/mm^3^, hyperferritinemia (2800ng/ml), elevated CRP (380mg/l), elevated fibrinogen levels (7.3g/l). She was intubated on the fifth day of admission, protective mechanical ventilation was initiated. Sedation was discontinued after 8 days and weaning was started. We noted prolonged weaning with unsuccessful spontaneous breathing trials. Tracheostomy was performed after 22 days after the onset of symptoms and 16 days of mechanical ventilation. Weaning of mechanical ventilation was successful after 5 days of tracheostomy. The patient´s comfort and her ability to communicate was improved. SARS-COV-2 PCR was positive the day of tracheostomy.

**Case 5:** a 76 years old, with known history of diabetes and hypertension, was admitted for ARDS due to SARS-COV-2 infection. Chest tomography showed ground glass opacities and images of consolidation. On admission, the patient was conscious with a GCS at 15/15, respiratory rate was 35 cycles per minute, pulse oximetry was 84%. Blood pressure was 180/100mmHg and heart rate 110 beat per minute. Oxygen therapy using a high-concentration mask was initiated and peripheral intravenous line secured. Hydroxychloroquine and azithromicyne were initiated. Biology revealed a lymphocyte account at 1230/mm^3^, ferritinemia at 608ng/ml, an elevated CRP at 250mg/l, fibrinogen at 1.5g/l. The patient was intubated 48 hours later. Clinical and biological improvement was noted after 10 days of mechanical ventilation, then the patient was extubated after 14 days of mechanical ventilation. The patient was then put on non-invasive ventilation. Due to persistant hypoxemia, re-intubation was performed after 5 days. Tracheostomy was performed after 28 days of the onset of symptoms and after a total of 20 days of invasive mechanical ventilation. Weaning was sucesssful after 7 days of tracheostomy and respiratory support was no longer nessecary. The patient is still in our ICU for nutritional rehabilitation and physical therapy. SARS-COV-2 PCR was positive the day of tracheostomy. It turned negative 10 days later.

## Discussion

The COVID-19 pandemic caused by the new coronavirus “SARS-COV-2” is a new challenge for ICU physicians. Although, this disease is associated with low mortality rates, it is considered to be highly contagious. In fact, COVID-19 is transmitted through aerosol and fine droplets and medical staff performing aerosol generating procedures is at high risk of contamination. Therefore common ICU procedures are completely transformed and rapidly evolving guidelines are focusing on staff safety during aerosol generating procedures. COVID-19 patients require ICU admission and respiratory support in 20-30% of cases. Prolonged weaning and unsuccessful extubation´s rates are high among them. Tracheostomy is proposed as a procedure that may reduce mechanical ventilation´s duration and may lead to shorter ICU stay. In fact, management of secretions and suctioning is made easier. Patient´s comfort is improved and therefore the use of heavy sedation and paralytic agents is decreased. Furthermore, ventilator weaning support, physical therapy are improved due to the reduction of dead space. However, tracheostomy is a highly aerosol generating procedure and multiple guidelines would not recommend it before 2 to 3 weeks after intubation.

Regarding the timing of tracheostomy, the New York head and neck society recommends waiting until approximately 21 days after the onset of symptoms in order to avoid exposing health care teams to increased risk of contamination [1]. Current literature also suggest that tracheostomy should only be considered after 14 days of invasive mechanical ventilation when the patient is still not suitable for extubation [2]. Additionally, it is preferable to wait for negative “SARS-COV-2” PCR testing whenever possible before considering tracheostomy [3]. In our series, tracheostomy was performed after an average of 24 days after onset of symptoms and after an average of 15 days of mechanical ventilation. All patients presented with prolonged and difficult weaning and 3 of them had unsuccessful extubation due to cough inefficacy and abundance of secretions. Tracheostomy was performed as part of weaning process in one case, as difficult weaning was predictable. Respiratory support was no longer necessary 5 days later. We followed the ENT-UK instructions on how to safely perform surgical tracheostomy in COVID-19 patients.

**Figure 1:**
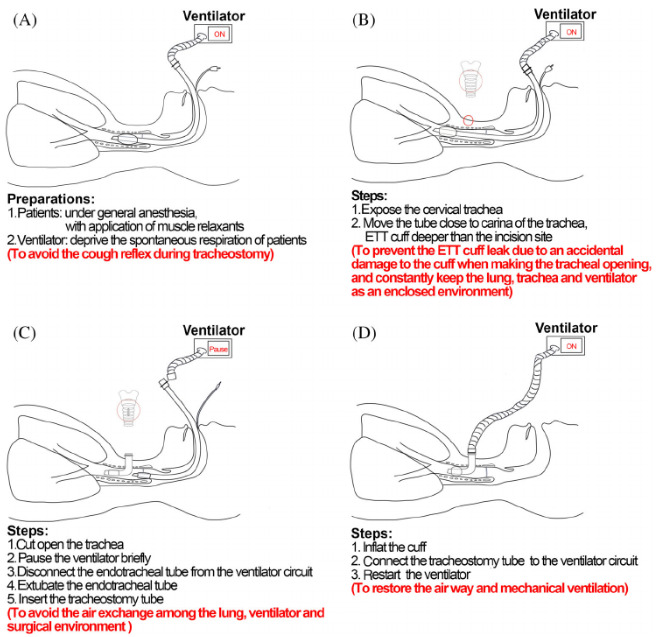
The optimized procedures of tracheostomy step by step for COVID-19 patients

We have decided to organize the procedure into 4 domains: ICU setting: all tracheostomies were performed at the bedside in ICU rooms. Personal protective equipment (PPE) for a Covid-19 surgical tracheostomy comprised a FFP2 face mask, surgical hood, goggles or visor and double gloves. Operating table, tracheostomy´s surgical tools and cannula are checked in advance; Tracheostomy team: the same operators intervened in all tracheostomies: senior ICU doctor: performing tracheostomy; senior ICU doctor: managing the airway and Ventilator settings during the procedure; junior ICU doctor: as a surgical assistant; anesthesiology and ICU nurse: managing general anesthesia and neuromuscular blockage during the procedure. Pre-tracheostomy: we performed:deep suctioning of the chest using the closed suctioning circuit; oral cavity suctioning; positioning of the patients. Tracheostomy: surgical tracheostomy was performed in all cases. We didn´t use monopolar nor bipolar diathermy through the procedure as they are considered to be aerosol-generating techniques. During the procedure, we followed steps on how to avoid nosocomial spread during tracheostomy for COVID-19 patients published by Xiao et al (Figure 1) [4]. SARS-COV-2 PCR was positive in all of our patients the day tracheostomy was performed, however, none of our tracheostomy team members has developed any symptoms of fever, general malaise, cough, shortness of breath and/or have tested positive for COVID-19.

## Conclusion

Surgical tracheostomy was successfully performed in 5 COVID-19 patients. Sedation was consequently decreased in all cases and weaning from mechanical ventilation was successful in one case. Our Tracheostomy team members were not contaminated.

## Competing interests

The author declares no competing interests.
